# Biomarkers in the IL-6 Pathway and Cardiovascular Outcomes in Patients With STEMI in LATITUDE-TIMI 60

**DOI:** 10.1016/j.jacadv.2024.100866

**Published:** 2024-02-22

**Authors:** David D. Berg, Michelle L. O'Donoghue, Petr Jarolim, Jianping Guo, Marc S. Sabatine, David A. Morrow

**Affiliations:** a TIMI Study Group, Brigham and Women’s Hospital, Harvard Medical School, Boston, Massachusetts, USA; b Brigham and Women’s Hospital, Harvard Medical School, Boston, Massachusetts, USA

**What is the clinical question being addressed?**

What is the relationship between “upstream” intermediates in the IL-6 pathway and cardiovascular outcomes in STEMI?

**What is the main finding?**

TNF-α and IL-6 are independently associated with higher cardiovascular risk, and patients with persistently elevated TNF-alpha and IL-6 after STEMI have the highest risk.

Inflammation is central to the pathogenesis of atherosclerotic cardiovascular disease (ASCVD) and is further amplified in the setting of myocardial infarction (MI). C-reactive protein (CRP) is a downstream inflammatory protein that is associated with increased cardiovascular risk in stable ASCVD and acute coronary syndromes, including ST-segment elevation myocardial infarction (STEMI).^1^ Given these links, upstream proteins in the central immune pathway linking interleukin (IL)-1, tumor necrosis factor (TNF) α, and IL-6 are of interest as potential targets for therapeutic interventions across the spectrum of ASCVD.^2^ In the CANTOS trial,^3^ anti-inflammatory therapy targeting IL-1β reduced cardiovascular events in patients with stable coronary artery disease, and in an ongoing trial (NCT05021835), anti-IL-6 therapy is being evaluated for cardiovascular protection in stable ASCVD. Whether directly targeting this pathway in acute coronary syndromes could mitigate the consequences of inflammation and translate to clinical benefit is of interest.

To gain further insights, we performed a nested biomarker substudy of patients with STEMI in the LATITUDE-TIMI 60 trial, a randomized, placebo-controlled trial that tested the efficacy and safety of the p38 MAP kinase inhibitor losmapimod in acute MI.^4^ The trial enrolled 865 patients with STEMI and 2,624 patients with non-STEMI. Although losmapimod-treated patients had lower CRP levels at 4 weeks following MI than did placebo-treated patients, there was no significant difference in the risk of major adverse cardiovascular events (MACE), defined as cardiovascular death, MI, or severe recurrent ischemia requiring urgent coronary revascularization, either in the overall cohort or in the prespecified subgroup of patients with STEMI.

For this analysis, we collected ethylenediaminetetraacetic acid plasma samples from patients with STEMI at enrollment (n = 855) and at 4 weeks postrandomization (n = 727). We measured TNF-α and IL-6 using enzyme-linked immunoassays (Ella ProteinSimple). We did not evaluate IL-1β since IL-1β levels are less reliably measured in plasma due to their low circulating concentrations. The clinical outcome for this analysis was MACE, the components of which were adjudicated by a clinical events committee according to standard definitions.

We evaluated the risk of MACE at 24 weeks according to baseline biomarker concentration (quartiles). We estimated the association of each biomarker with MACE using Cox regression, adjusting for age, sex, diabetes, smoking, estimated glomerular filtration rate, prior heart failure, prior stroke, presentation with anterior MI, peak troponin or CK-MB, high-sensitivity CRP, and randomized treatment. We performed an exploratory landmark analysis of clinical outcomes beginning at 4 weeks according to whether each biomarker was high (ie, above the median baseline value of the cohort) vs low at baseline and 4 weeks postrandomization. The ordinal risk of MACE across quartiles was assessed using the log-rank test for trend. Finally, we evaluated the treatment effect of losmapimod on each biomarker from baseline to 4 weeks postrandomization using an ANCOVA model,^4^ adjusting for the same clinical covariates.

The median age of the cohort was 66 years; 31% were women; 98% underwent cardiac catheterization; and 95% were on statin therapy. The median time from symptom onset to randomization was 3.8 hours (IQR: 2.5-6.6 hours), and study drug was administered at a median of 0.2 hours (IQR: 0.1-0.6 hours) prior to primary percutaneous coronary intervention.

**Figure 1:**
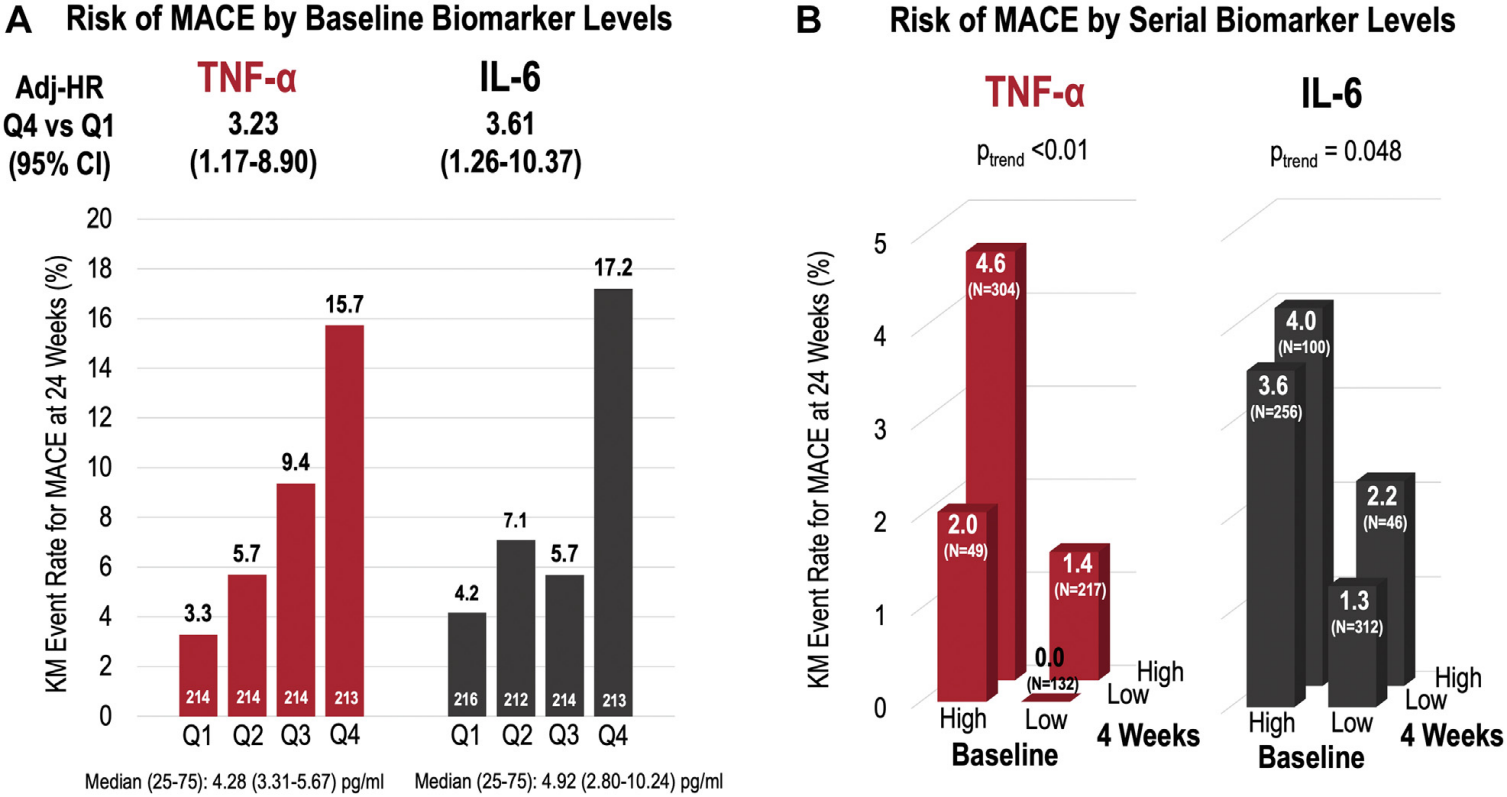
Inflammatory Biomarkers and Cardiovascular Risk (A) Risk of MACE by baseline biomarker levels. (B) Risk of MACE by serial biomarker levels. IL = interleukin; MACE = major adverse cardiovascular events; Q = quartile; TN = tumor necrosis factor.

In adjusted analyses, TNF-α (adjusted HR for top vs bottom quartile: 3.23; 95% CI: 1.17-8.90) and IL-6 (adjusted HR: 3.61; 95% CI: 1.26-10.37) were each independently associated with higher rates of MACE (Figure 1A). Furthermore, in a landmark analysis from 4 weeks through the end of follow-up (24 weeks), patients with high concentrations of TNF-α and IL-6 at both baseline and 4 weeks postrandomization had the highest rates of MACE (Figure 1B). Losmapimod did not significantly modify concentrations of TNF-α (ratio of the geometric means for losmapimod vs placebo, 0.93; 95% CI: 0.83-1.05; *P* = 0.25) or IL-6 (0.99; 95% CI: 0.85-1.16; *P* = 0.89) between baseline and 4 weeks postrandomization.

In this nested biomarker study from LATITUDE-TIMI 60, TNF-α and IL-6—2 inflammatory proteins in the central IL-6 signaling pathway—were independently associated with a higher risk of MACE in patients with STEMI, and patients with persistently elevated TNF-α or IL-6 levels 4 weeks after STEMI had the highest risk. Although TNF-α and IL-6 were not significantly modified by losmapimod in patients with STEMI, these data raise the possibility that other agents directly targeting upstream intermediates in the IL-6 signaling pathway may have therapeutic potential in the treatment of STEMI.
